# Feasibility of delivering foot and ankle surgical courses in a partnership in Eastern, Central and Southern Africa

**DOI:** 10.1186/s12909-022-03142-y

**Published:** 2022-02-04

**Authors:** R. R. Brown, M. B. Davies, G. Drury, J. Lane, C. Lavy, S. Nungu, J. Munthali

**Affiliations:** 1 University of Oxford, Oxford, UK; 2 University of Sheffield, Sheffield, UK; 3 Muhimbili National Hospital, Dar es Salaam, Tanzania; 4 University of Zambia, Lusaka, Zambia

**Keywords:** Training, Surgery, BOFAS, Africa, COSECSA, Courses, Foot, Ankle

## Abstract

**Background:**

Foot and ankle pathology if not treated appropriately and in a timely manner can adversely affect both disability and quality adjusted life years. More so in the low- and middle-income countries where ambulation is the predominant means of getting around for the majority of the population in order to earn a livelihood. This has necessitated the equipping of the new generation of orthopaedic surgeons with the expertise and skills set to manage these conditions. To address this need, surgeons from the British Orthopaedic Foot & Ankle Society (BOFAS) and College of Surgeons of Eastern, Central and Southern Africa (COSECSA) transferred the “Principles of Foot and Ankle Surgery” course to an African regional setting. The course was offered to surgical trainees from 14-member countries of the COSECSA region and previously in the UK. The faculty was drawn from practicing surgeons experienced in both surgical education and foot and ankle surgery. The course comprises didactic lectures, case-based discussions in small groups, patient evaluations and guided surgical dissections on human cadavers. It was offered free to all participants. The feasibility of the course was evaluated using the model defined by Bowen considering the eight facets of acceptability, demand, implementation, practicality, adaptation, integration, expansion and limited efficacy. At the end of the course participants were expected to give verbal subjective feedback and objective feedback using a cloud based digital feedback questionnaire. The course content was evaluated by the participants as “Poor”, “Below average”, “Average”, “Good” and “Excellent”, which was converted into a value from 1–5 for analysis. The non-parametric categorical data was analysed using the Two-sample Wilcoxon rank-sum (Mann–Whitney) test, and significance was considered to be *p* < 0.05.

**Results:**

Six courses in total were held between 2018 and 2020. Three in the UK and three in the COSECSA region. There were 78 participants in the three UK courses and 96 in the three courses run in the COSECSA region. Hundred percent of the UK participants and 97% of the COSECSA participants completed the feedback. Male to female ratio was 4:1 for the UK courses and 10:1 for the COSECSA Courses. In both regions all the participants responded that they would recommend the course to their colleagues. Among the COSECSA participants 91% reported that the course was pitched at the right level, which is similar to the 89% of the UK participants (*p* = 0.28).

**Conclusion:**

The BOFAS Principles of Foot and Ankle Surgery course design provides core knowledge, with an emphasis on clinical examination techniques of the foot and ankle, while at the same time, caters for the anticipated difference in the local clinical case mix and resources. This study establishes that by attending the course surgical trainees can achieve their learning goals in foot and ankle surgery with the same high quality qualitative and quantitative feedback in both regions. This would improve their clinical practice and confidence. The multifaceted approach adopted in this course blending didactic teaching, small group discussions, interactive sessions, case-based discussions, cadaveric surgical skills training printed educational materials and feedback helped fulfil these educational objectives. Working in partnership with local expert orthopaedic surgeons from a number of Sub-Saharan countries, was key to adapting the course to local pathology and the COSECSA setting.

## Background

The Lancet Commission on Surgery highlighted a need for surgical services in low and middle income countries (LMICs) to scale up [1], in order to address many major challenges, including from traumatic injuries in the developing world. This requires teaching a new generation of surgeons the surgical skills to manage the full width of injuries [2]. Trauma involving the foot and ankle accounts for 20% of all adult fractures in the under 40 year olds in LMICs [3]. Infection, degeneration as well as both neurological and persistent childhood conditions add to the burden of unmanaged foot and ankle pathology. Therefore the growing cohort of Orthopaedic Surgeons in Africa will need better awareness and training in clinical and surgical skills of Foot and Ankle Surgery.

The teaching of the relatively new sub-specialty of Foot and Ankle surgery has lagged behind other orthopaedic sub-specialties. In 2011, the British Orthopaedic Foot & Ankle Society (BOFAS) designed the “Principles of Foot and Ankle Surgery” course. This course was aimed at experienced orthopaedic surgical trainees in the UK and initially evolved in response to participants’ feedback. A central philosophy of the course is plentiful interaction with an approachable faculty, which is achieved with a low faculty to student ratio. Seventeen didactic lectures teach the key topics of foot and ankle surgery, while other teaching methods include; small group teaching of clinical examination skills of both normal and pathological feet of patients; case based discussions and a cadaveric dissection session. The dissection training refreshes the participants’ knowledge of surgical anatomy and allows practice of surgical approaches.

With the expansion and improved co-ordination of surgical training in Africa, similar high quality training must be designed and delivered to improve the preparedness of surgeons in managing foot and ankle pathologies. In co-operation with COSECSA (The College of Surgeons of East, Central and Southern Africa), BOFAS aimed to offer the same “Principles of Foot and Ankle Surgery” course to a generation of COSECSA trainees. This paper describes the learning points from the partnership which aimed to deliver the same high quality course in a different setting. Our hypothesis is that it is feasible to use the same training methods to deliver the same course in a partnership and obtain the same high quality feedback in the COSECSA region.

## Method

### Course design

**Fig. 1:**
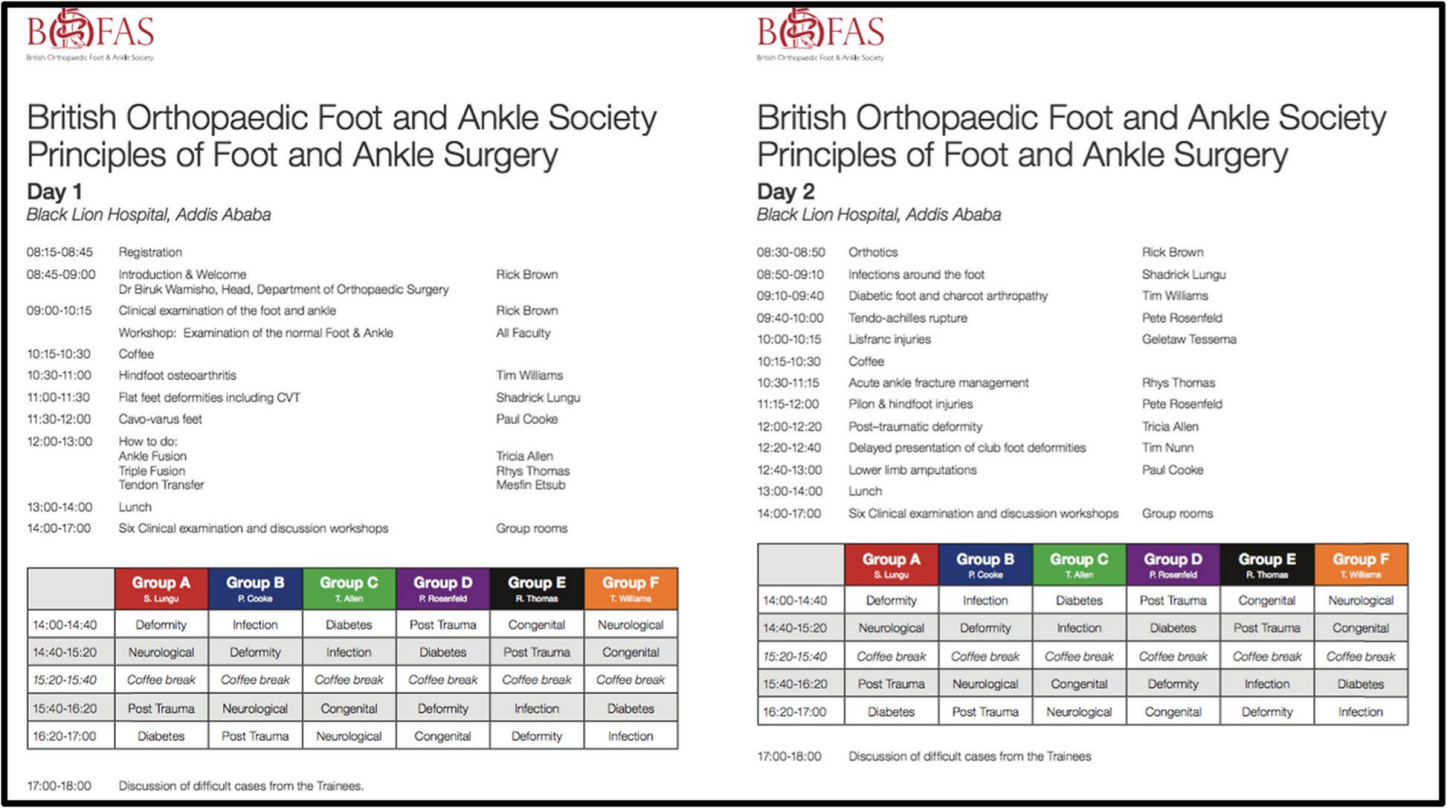
The Timetables of UK & COSECSA Courses

The well established BOFAS Principles of Foot & Ankle Surgery is a three day course which aims to teach the skills to examine the foot and ankle, to construct a differential diagnosis, to interpret clinical signs and to use higher order thinking to make a management plan. In both regions the course consists of a core curriculum covered in the first two days and a cadaveric dissection done on the third day. Didactic lectures were given each morning with small group teaching of clinical examination skills with patients in the afternoons, followed by case based discussions in the late afternoon (See Fig. 1).

The course timetable in both the UK and COSECSA, starts with a comprehensive lecture on the clinical examination of the foot and ankle, followed by an interactive session, in which the participants examine each other’s feet to establish a system of assessing a “normal” foot. This system of examination and thinking is reinforced throughout the first two days. On each of the first two afternoons, the host hospital organises patients to attend with foot and ankle pathologies. Finally the case based discussions re-explore the knowledge from the didactic lectures and allow the participants to reinforce their understanding.

**Fig. 2:**
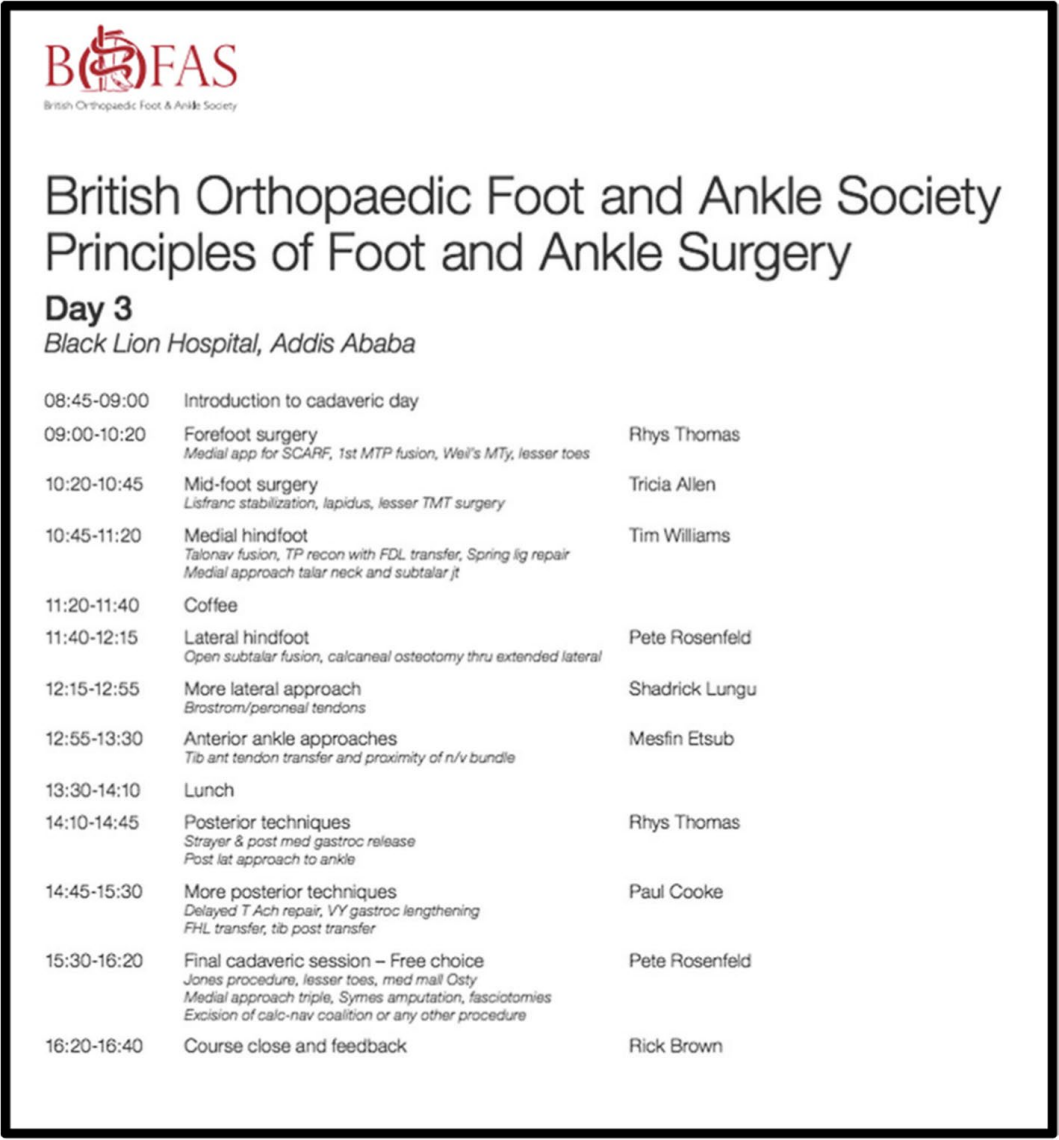
Timetable of Third Day of Dissection Training

The third day occurs in the anatomy dissection room (See Fig. 2) and consists of a series of short mini-lectures, each of five minutes duration, followed by sequential dissection of the human cadavers to refresh surgical anatomy and to practice surgical approaches. In the UK, improved access to fresh-frozen cadaveric specimens has transformed the teaching of surgical anatomy because of the more realistic tissue-handling properties of cadaveric specimens preserved in this manner. In the COSECSA region, there is still limited access to cadaveric specimens preserved in this manner. A foot specimen preserved with the traditional use of formalin, has less pliable tissue, making demonstration of tissue handling more difficult. Each faculty surgeon taught no more than four participants with two lower limb specimens and was able to demonstrate anatomical structures, surgical approaches, as well as practical tips and tricks.

All participants were trainee surgeons on a recognised training programme in either the UK or a COSECSA member country. In both regions a minority of participants were working independently, as either a newly appointed consultant surgeon in Africa, or a non-training staff grade surgeon in the UK. All courses were available by open online application. The majority of participants were from the local training programme, but some places were taken by trainees from across the UK training system, or across the COSECSA training system. Thus in Africa participants came from Zambia, Ethiopia, Tanzania, Zimbabwe, Kenya, Malawi and Sudan.

The courses were run between January 2018 and March 2020. The course was free to all participants on all six courses and paid for by BOFAS with sponsorship from the Orthopaedic Industry.

### Design process for the modification of the course for COSECSA

A wide spectrum of experts were involved in the partnership to select the topics of the lectures and the choice of knowledge to be presented within each lecture or workshop. Key informants were involved from the University Teaching Hospital, Lusaka, Zambia, The Black Lion Hospital, Addis Ababa, Ethiopia and Muhimbili Hospital, Dar es Salaam, Tanzania, as well as those from the Universities of Oxford, London and Sheffield in the UK. Advice was given by representatives from the BOFAS Education Committee and the COSECSA Orthopaedic Board of Examiners.

Although the core components of the “Principles” course are common to both the UK and COSECSA courses; some components of the COSECSA course were modified to cater for the different range of pathology in the local population and the variation of surgical procedures readily available in each region. For example, the UK course has considerable time spent in discussing ankle arthroscopy or the treatment of ankle arthrosis by total ankle replacement. These topics were removed, as this surgery is not currently widely available in the COSECSA region. These were replaced by lectures on topics more pertinent to the region, such as infections of the foot and ankle, or the management of fractures and dislocations that present late. Foot & Ankle conditions taught within the paediatric orthopaedic syllabus, such as the early management of club feet were not included in either region.

### Training the trainers

For all courses, the faculty were selected, because they were practicing surgeons experienced in both surgical education and foot & ankle surgery. The majority were from the BOFAS Education Committee and were familiar with the concept and philosophy of the course having previously taught on at least two similar courses. Local regional faculty were introduced to the teaching styles of the course in a pre-course briefing and with online assistance to write their own presentations. The course faculty to participant ratio was 1:3 in UK and 1:5 in Africa.

### Feasibility assessment

In order to study all the relevant factors of the course and to determine whether it achieved its goals in the COSECSA region, we evaluated the course using an established model defined by Bowen [4]. This includes the eight facets of acceptability, demand, implementation, practicality, adaption, integration, expansion and limited efficacy.

### Participant assessment of the course

On completion of the course, participants gave verbal subjective feedback in an open discussion exercise and they submitted objective feedback into a cloud based digital feedback questionnaire, in order to receive a course completion certificate. During the same time, between January 2018 and March 2020, the same data was collected and analysed after three consecutive BOFAS “Principles” courses in each region. Feedback on the content of the common core lectures was compared. The participants recorded their choice between “Poor”, “Below average”, “Average”, “Good” and “Excellent”, which was converted into a value from 1–5 for analysis. Participants were asked questions to assess whether their training goals had been achieved and whether it would improve their clinical practice. The responses of the participants to the three interactive sessions, which employed three different teaching styles, were assessed. There was no testing of the post-course knowledge of the participants, in either the UK or in the COSECSA region.

### Data management and analysis

The non-parametric categorical data was analysed using the Two-sample Wilcoxon rank-sum (Mann–Whitney) test. Significance was considered to be < 0.05. Each candidate gave a score of between 1–5, and therefore it was considered that a difference in the total scores of 10 was considered of sufficient size to determine a meaningful difference between the two groups. The power calculation confirmed there was an 80% likelihood of determining a 10 point difference between the 2 groups.

### Ethics

No patient identifying data was used in the courses and no aspects of the patients have been analysed or presented. The participants gave informed consent for their feedback to be anonymised and analysed. All regulations of the host University pertaining to human cadavers were followed.

## Results

### Participation

There were 78 participants in the three UK course, with 28 in Newcastle, 22 in Exeter and 28 in Oxford (mean 26); while there were a total of 96 participants in the three courses undertaken in the COSECSA region, with 22 in Zambia, 34 in Ethiopia and 40 in Tanzania, (mean 32). 97% of COSECSA participants and 100% of UK participants completed the feedback. The Male: Female ratio of participants was 4:1 in the UK courses and 10:1 for the COSECSA courses. Although free to each participant, the cost to the organisers per participant was £311 in Zambia, £294 in Ethiopia, £377 in Tanzania and £644 in the UK.

### Feasibility assessment: acceptability

Between the two continents. There was no difference in the distribution of participants who reported that they had met their learning objectives, and that the course would improve their clinical practice (*p* = 0.51). In both regions all the participants would recommend the course to colleagues. The participants scored the content of the core lectures very highly in both regions (Table 1). This would suggest that the choice to include these topics in both the UK and the COSECSA region was justified. On close inspection the COSECSA surgeons were less complimentary (60% excellent) about the content within the diabetes lecture, than the UK trainees (77%). This may reflect the lower prevalence of diabetes, which is a very contemporary topic in western medicine with its increasing burden on healthcare systems. Although with the increasing incidence of diabetes in Africa, the knowledge of diabetic foot complications will become more important.

**Table 1:** The Score from the Participants for the Content of the Core Lectures

	**UK (*n* = 78)**					**COSECSA (*n* = 96)**					*p*-value
	Excellent	Good	Average	Below average	poor	Excellent	Good	Average	Below average	poor	
**Core Lectures**											
Clinical examination of the normal foot and ankle	81%	19%	0%	0%	0%	72%	22%	2%	0%	0%	0.51
The neurological cavovarus foot	76%	23%	1%	0%	0%	66%	28%	2%	0%	0%	0.28
Diabetes in the foot and ankle	77%	18%	5%	0%	0%	60%	37%	3%	0%	0%	0.38
Orthotics	63%	26%	11%	0%	0%	67%	30%	1%	1%	0%	0.51
Management of ankle fractures	82%	17%	1%	0%	0%	78%	21%	1%	0%	0%	0.51
Hindfoot trauma	79%	19%	1%	0%	0%	67%	32%	1%	0%	0%	0.51

**Fig. 3:**
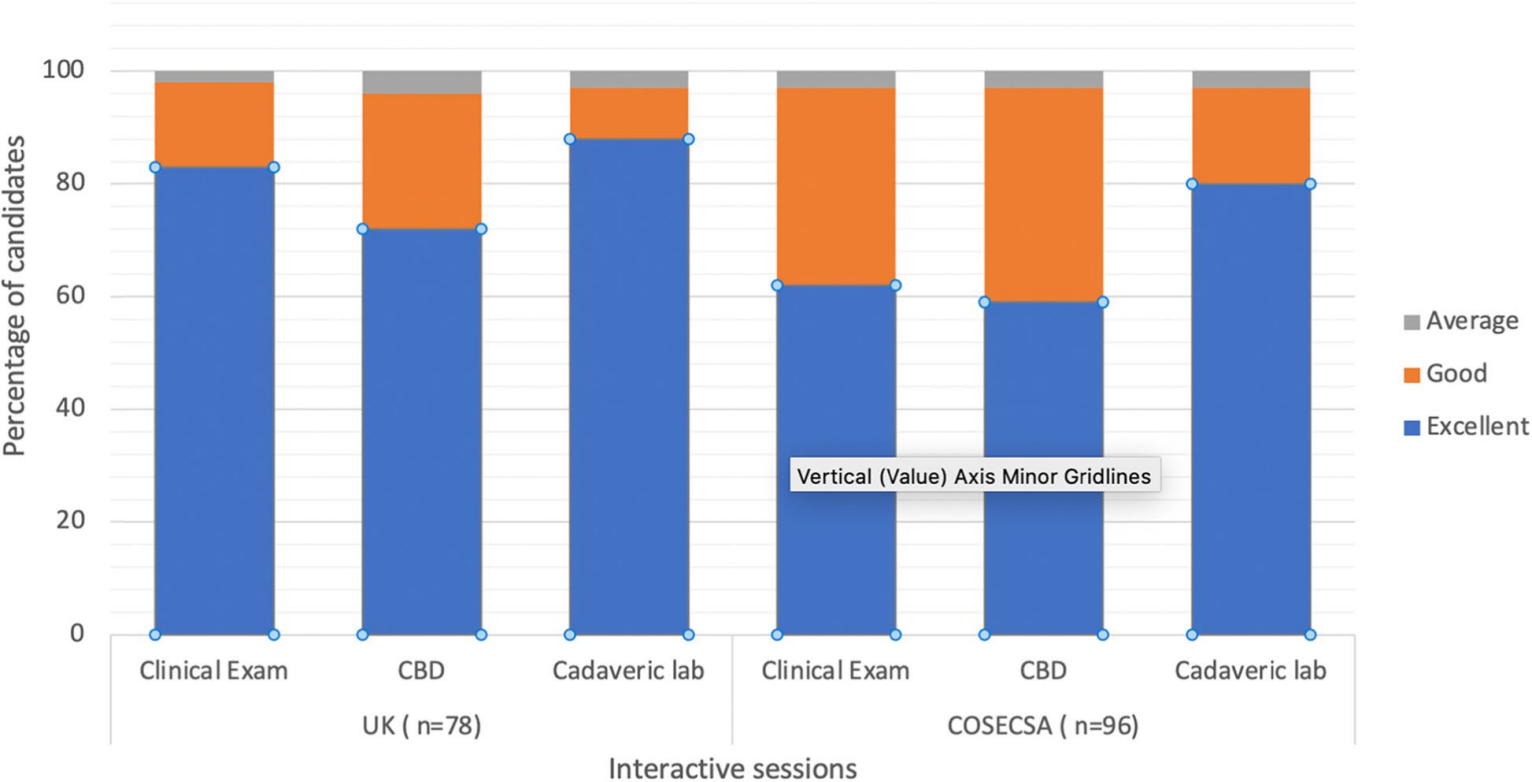
The Distribution of Feedback on the Three Interactive Teaching Methods

**Fig. 4:**
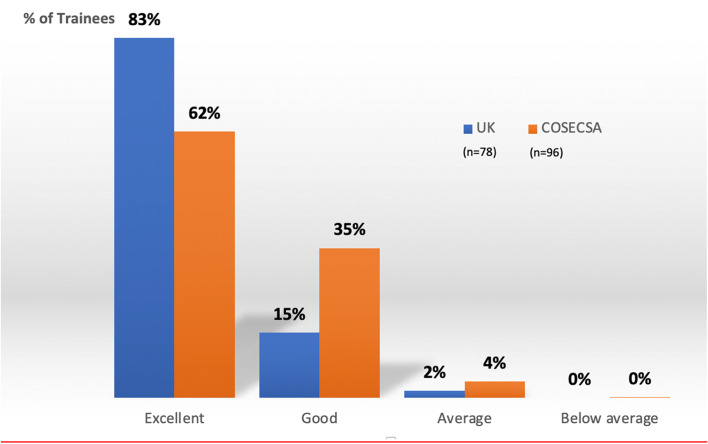
The Comparison of the Feedback from the Small Group Session on Clinical Examination Skills

There were three interactive teaching styles, which were small group teaching of clinical examination skills, case based discussions (CBDs) and cadaveric workshops. The participants scored all three teaching styles highly ( see Fig. 3). The core skill for the participant to learn is a system to examine the Foot & Ankle and it was therefore important to see that 97% of COSECSA trainees reported this aspect of the training as Excellent/Good (Fig. 4). There was no significant difference in the feedback between the between the UK and COSECSA trainees. The cadaveric workshops are universally acceptable and popular with surgical trainees.

### Demand

Ninety one percent of COSECSA participants reported that the course was at the right level, which is similar to the 89% of the UK participants (*p* = 0.28). The distribution of participants who felt more confident about safe practice, in foot and ankle surgery was the same in both groups, with 100% of UK trainees and 98% of COSECSA trainees reporting Excellent/Good. Each successive course in Africa has recruited participants more quickly, as the popularity circulates on the social media of surgical trainees. In the UK each principles course is over subscribed within one week of opening.

### Implementation

Some UK faculty members reported to the Course Director an initial difficulty establishing the level of experience of the audience on the first day. However this level was established during the interactive sessions. The faculty reported that after the third Principles course the appropriate level of the didactic lectures had been established and a library of the core lectures has been created for use by future faculty members. This is similar to the UK Principles course. The role of any Course Director is always key to the smooth flow of a course. This was especially challenging with this course in Africa and a Course Directors Instruction Manual training book is now available.

### Practicality

This training required adequate space**,** audio-visual equipment, ventilation, accommodation and refreshments. The patients required respectful, care, transportation and refreshments. Without the co-operation of the patients the key learning objective of clinical examination cannot be taught. A language barrier often occurred if the small group of participants did not contain a local trainee. The local organising committee had to select patients with good clinical signs from a short list of foot & ankle pathologies. Their support role was very important.

Approachability of the faculty is a key philosophy of the course and barriers were broken down by the supportive atmosphere of the small group teaching and the traditional course dinner to breakdown the formality.

Cadaver training requires advanced storage facilities for the specimens with safe practices in infection control and respectful disposal. These facilities are starting to become more widely available in the COSECSA region.

### Adaption

During the three renditions of the course in Africa, each lecture was subtly improved and adapted by the next lecturer, in order to become more appropriate to the local resources and pathologies. The key surgical principles remained the same, but were often applied to local pathology. Topics were omittedif already taught elsewhere, such as the closed management of foot and ankle fractures, taught by the AO- Alliance [5]. High tech treatments were not included within didactic lectures. Interestingly in the open questions, the participants requested to know the state of the art managements in Europe, even when these facilities were not yet widely available in Africa.

### Integrations

The management of foot & ankle problems has become more integrated with the COSECSA syllabus, due to the closer working of BOFAS and COSECSA surgeons. New examination questions have been written, which will further fuel the desire of future surgical trainees to learn the principles of foot & ankle surgery.

### Expansion

In 2019 35 trainees complete the Othopaedic Fellowship of COSECSA. This had increased 120% over the three preceding years and a similar future expansion is predicted. Additional orthopaedic surgeons complete the local exit exams in their own country. Thus the need for this type of structed surgical training will expand.

### Limited efficacy

The trainees are observed to be able to examine and interpret clinical findings, as well as discuss cases in a controlled environment. But we have not shown whether this applies to when they return to a busy hospital in their home environment. The participants are able can display anatomy on the cadaver, where there is no bleeding. These skills will help in real surgical procedures, but cannot be performed if there is a lack of available equipment.

## Discussion

### The requirement for the course and the partnership

A lack of confidence in the management of foot and ankle conditions was noted by the senior authors at the Fellowship of the COSECSA Orthopaedics examinations in Mombasa, Kenya in 2016. Indeed a review of feedback from Zambian orthopaedic trainees reported challenges in obtaining training in procedures, such as foot and ankle surgery, which were outside of the core specialties, and that without obtaining specialized training they had difficulty following textbook surgical practice in less common more difficult cases [6]. Similarly until the British Orthopaedic Foot and Ankle Society (BOFAS) developed the “Principles of Foot and ankle surgery” course, there was a perception from UK trainee surgeons towards the end of their training were underprepared in the fundamentals of foot and ankle surgery. From 2011, over 600 surgeons have attended 25 UK courses. Although the principles of the course have remained very consistent, the course content evolved over the first seven year in response to participant feedback, which has been consistently good.

Therefore it was proposed to transfer this training course into the COSECSA region. The BOFAS Council agreed to support and fund a pilot course at the University Teaching Hospital, Lusaka, Zambia in April 2018. After reviewing the first feedback, another three annual courses were funded by BOFAS.

The lack of surgical training courses in Africa has been considered as a “major barrier” in access to a health care provider, as reported by Smythe for the treatment of clubfeet[7]. In Africa, a transformative scale-up of medical education is needed which will need both increased capacity and better co-ordination between the health and the education sectors. Educational institutions are not necessarily linked to national health systems or health delivery systems, which can limit how doctors learn or maintain their surgical skills [2]. COSECSA co-ordinates surgical training across many educational and health care organisations in many countries. Thereby a partnership with COSECSA provides a great outreach for a foot and ankle surgical course.

### Feasibility and strengths of the course

This study shows that the course design was both acceptable and fulfilled an educational demand. It provides core knowledge, with an emphasis on clinical examination techniques of the foot and ankle, while at the same time, catered for the anticipated difference in the pathology and local clinical case mix. There is a strong emphasis on trauma in the didactic lectures, case based discussions and in the selection of patients for examination practice. The COSECSA participants confirmed the correct level of content of the trauma lectures, for example, with 78% participants scoring the ankle fracture lecture as excellent.

Sub-Saharan Africa has one of the highest mortality rates for trauma, with an individual being almost 14 times more likely to die following road traffic collisions than in the UK [8]. This burden of mortality from trauma is likely to surpass both AIDS and TB in the coming decade. The morbidity in the survivors of trauma is a second major burden, which is especially a problem after high energy foot and ankle injuries. The geographical distribution of trained surgeons in Africa is imbalanced, which can lead training to have an urban emphasis, rather than focusing on the more common conditions found in rural practice. This syllabus emphasised common foot & ankle treatments such as ankle fractures, over highly technical treatments available only in the major cities.

The review of the implimentaion of the course has shown that surgical trainees in both regions can achieve their learning goals in foot & ankle surgery by attendance. Both groups reported equally that the course improved their clinical practice and confidence. Regardless of location, the course was unanimously given the accolade of being recommendable to a colleague. This may only be a reflection of a general gratitude to have any instruction in this sub-specialty area. All four types of teaching techniques worked with the COSECSA trainees.

The integration of local and British faculty allowed a breadth of clinical experience of foot & ankle pathologies from both regions. The selection of enthusiastic approachable faculty is vital. Small group teaching is used to teach the key skill of how to examine a foot & ankle. The approachability of the faculty is essential, and this was effective at teaching trainees in both regions. Case based discussions allow focused learning and were used to highlight the earlier learning from the didactic teaching. It was interesting that a smaller percentage of COSECSA trainees recorded this as excellent, which may be explained by less exposure to this technique, a traditional respect for visiting experts or perhaps other cultural factors.

The challenging practicalities of delivering human cadaver training were worthwhile. COSECSA surgical trainees were the same as trainees across the world and loved learning surgical approaches in a cadaveric workshop. In addition to seeing the anatomy exposed correctly, surgery on a cadaver allows the trainee to explore the neighbouring neuro-vascular structures. The high satisfaction justified the greater practical difficulties and costs incurred in running this interactive third day in an African hospital. Important regulations must be fulfilled about sourcing and preserving the cadavers. The freshly thawed previously frozen cadavers are the best for demonstrating surgery but are more difficult to obtain.

A multi-faceted strategy, combining didactic teaching, small group sessions, interactive workshops, case based discussions, local opinion leaders, printed educational materials and feedback, has been shown in the general educational literature to better fulfill educational objectives [9]. This range of teaching methods contributes to the success of the BOFAS “Principles of foot and ankle surgery” and were further tailored to the specific setting. This multi-faceted strategy has been shown to aid the teaching of treatments for club foot deformities [10].

Although modern video conferencing and webinar technology, can transmit knowledge between surgeons across the world. the teaching of tactile skills such as the clinical examination of joints and operative technique still requires in person interaction. The teaching of surgical skills remotely is in its infancy. A case controlled study showed an improvement in the skill of Obstetric Trainees in Makerere University, Kampala, Uganda, to tie a surgical knot, after remote teaching from the University of California. [11]. With the global COVID 19 pandemic on line education has accelerated, but for now the teaching of clinical examination and surgical skills need this type of face to face interactive course.

### Limitations of the course

The original UK course aimed to full fill the curriculum to accomplish the UK Certificate of Completion of Training (CCT). Although considerable thought was applied as how to involve local pathologies from the African setting, there is not yet a formal detailed COSECSA syllabus to match. Prospectively a more in depth syllabus is being developed for the FCS (ORTH) COSECSA exam against which the learning goals could be measured.

The course with COSECSA had a maximum of eight trainees to a faculty member in the interactive teaching, which was larger than the six participants on the UK course, which may have had a deleterious effect on some of the feedback. A balance was made between reaching as many trainees as possible without diluting the individual experience. However Dreyer concluded in a study of teaching surgical skills in general surgical emergencies, that eight participants per tutor was too large a ratio to be of maximal educational benefit and advocates just six participants [12]. This slightly smaller group size would have a major financial implications, and would need justification when obtaining longer term funding.

These initial courses had the environmental and financial disadvantages of requiring a minimal faculty to travel from the UK. Our course tries to use the cascade concept to expand the regional COSECSA faculty of foot and ankle surgeons and reduce the dependency on travelling British faculty, which will allow an expansion in the capacity of courses each year. These “cascade style” courses have been effective at educating a broad spectrum of healthcare workers in trauma and orthopaedic topics in the COSECSA region [13].

Future courses in Africa could be supported by tutorials using video conferencing by surgeons in the UK, who have previously taught in the COSECSA region and understand the local pathologies.

Many lessons have been learnt which will help the modification and transfer of other surgical courses to low and middle income countries as we train the next generation of surgeons to address the global burden of surgical disease.

### Lessons learnt

1. A course focused on the Principles of Foot & Ankle Surgery can be successfully transferred from the UK to the Sub-Saharan African setting.
2. Partnership is key to modify and develop the course content with expert local Orthopaedic Surgeons who are familiar with the pathology and surgical resources of the region.
3. The interactive teaching techniques of case based discussion groups, small group teaching of examination skills and training in surgical approaches with cadaver surgery, work very effectively. They justify the additional funding and practical challenges of a small participant to faculty ratio.
4. Collaborating with the established Training system of the College of Surgeons of East, Central & Southern Africa improved the reach of the course.
5. The cascade method of training COSECSA regional faculty will allow expansion of future course capacity.

### Limitations of this study

No assessment of participant knowledge was conducted. The faculty and trainees were together for just three days in the host country and the precious time was used to impart rather than test knowledge. With more time, a question-based assessment with a pass/fail competency would be beneficial, or a digital system could be conducted remotely.

The small numbers of participants in each course limited our ability to demonstrate any variation in learning and feedback across different sites, or any time effect as the delivery of the course in Africa was expected to have improved each time.

One key limitation was no further feedback was sought at a later time point. Other trauma courses in this region have confirmed that learning has led to a change in practice, by repeating the assessment of feedback six months after the end of the course [14]. However the ongoing use of these new skills was reported in this illustrative comment from The Course Director in Dar es Salaam made one year after the course,

“Thanks very much for the Foot & Ankle course because we have marked a great change in the management of our patients. Doctors are very active and are handling the once neglected or mismanaged cases well as we had wished with this course. So thank you.”

## Conclusions

In a partnership between COSECSA and BOFAS, The Principles of Foot & Ankle surgery course was successfully able to teach Orthopaedic Surgical trainees from a range of Sub-Saharan countries, and obtain equally high quality qualitative and quantitative feedback, similar to that received from their counterparts in the UK. Although the core principles remain consistent, adjustments can be made for local pathology by partnership in a consultative process with local surgical trainers. The approachability of an enthusiastic multi-national faculty and the small participant to faculty ratio led to high quality training, which successfully employed a wide range of didactic and interactive teaching methods, to impart clinical examination and surgical skills.

## Data Availability

The dataset supporting the conclusions of this article is included within the article.

## References

[1] Meara JG, Leather AJM, Hagander L, Alkire BC, Alonso N, Ameh EA, Bickler SW, Conteh L, Dare AJ, Justine D (2015). Global Surgery 2030: evidence and solutions for achieving health, welfare, and economic developmen. Lancet.

[2] Celletti F, Reynolds TA, Wright A, Stoertz A, Dayrit M (2011). Educating a New Generation of Doctors to Improve the Health of Populations in Low- and Middle-Income Countries. PLoS Med.

[3] Pouramin, Panthea, et al. 2019 INORMUS Investigators A multicenter observational study on the distribution of orthopaedic fractures across low- and middle-income countries. OTA Int. 2019;2(3):e026. 10.1097/OI9.0000000000000026 PMC7997096 33937655

[4] Bowen DJ, Kreuter M, Spring B, Cofta-Woerpel L, Linnan L, Weiner D (2009). How We Design Feasibility Studies. American Journal of Preventive Medicine.

[5] Alliance, AO. AO Alliance - Educational Materials. [Online] 2020. https://ao-alliance.org/resources/publications/educationalmaterials/%23sf-%7B1:2018,2:English%7D.

[6] Freitas D, Munthali J et al. 2018 Surgical registrars' perception of surgical training and capacity in Zambia: Results from three COSECSA affiliated training hospitals. Am J Surg. pp. 744–751. 10.1016/j.amjsurg.2017.07.023 28764850

[7] Smythe T, Le G, Owen R, Ayana B, Hansen L, Lavy C (2018). The development of a training course for clubfoot treatment in Africa: learning points for course development. BMC Medical Education.

[8] Peter NA, Pandit H, Le G, Nduhiu M, Moro E, Lavy C (2016). Delivering a sustainable trauma management training programme tailored for low-resource settings in East, Central and Southern African countries using a cascading course model. Injury.

[9] Forsetlund L, Bjorndal A, Rashidian A, Jamtvedt G, O’Brien MA, Wolf F, et al. Continuing education meetings and workshops: effects on professional practice and health care outcomes. Cochrane Database Syst Rev. 2009;(2):Cd003030. 10.1002/14651858.CD003030.pub2 PMC7138253 19370580

[10] Smythe T, Owen R, Le G, Uwizeye E, Hansen L, Lavy C. The feasibility of a training course for clubfoot treatment in Africa: A mixed methods study. PLoS One. 2018;13(9):e0203564. 10.1371/journal.pone.0203564 PMC6136756 30212532

[11] Autry A, Knight S, Lester F (2013). Teaching surgical skills using video internet communication in a resource-limited setting. Obstet Gynecol.

[12] Dreyer J, Hannay J, Lane R (2014). Teaching the Management of Surgical Emergencies Through a Short Course to Surgical Residents in East/Central Africa Delivers Excellent Educational Outcomes. World J Surg.

[13] Peter NA, Pandit H, Le G, Nduhiu M, Moro E, Lavy C (2015). A multicountry health partnership programme to establish sustainable trauma training in east, central, and southern African countries using a cascading trauma management course model. Lancet.

[14] Ologunde R, Le G, Turner J, Pandit H, Lavy C (2017). Do Trauma Courses Change Practice? A Qualitative Review of 20 Courses in East. Central and Southern Africa. Injury.

